# The combination of four molecular markers improves thyroid cancer cytologic diagnosis and patient management

**DOI:** 10.1186/s12885-015-1917-2

**Published:** 2015-11-19

**Authors:** Federica Panebianco, Chiara Mazzanti, Sara Tomei, Paolo Aretini, Sara Franceschi, Francesca Lessi, Giancarlo Di Coscio, Generoso Bevilacqua, Ivo Marchetti

**Affiliations:** 1 Division of Surgical, Molecular, and Ultrastructural Pathology, University of Pisa and Pisa University Hospital, Via Roma 57, Pisa, 56100 Italy; 2 Department of Pathology, University of Pittsburgh School of Medicine, 200 Lothrop St, Pittsburgh, PA 15261 USA; 3 Pisa Science Foundation, Via Panfilo Castaldi 2, Pisa, 5612 Italy; 4 Sidra Medical and Research Center, Research Branch, Division of Translational Medicine, Al Corniche Street, PO 26999 Doha, Qatar; 5 Section of Cytopathology, University of Pisa and Pisa University Hospital, Via Roma 57, Pisa, 56100 Italy

**Keywords:** Thyroid cancer, Preoperative diagnosis, Indeterminate lesions, Molecular marker, Computational model

## Abstract

**Background:**

Papillary thyroid cancer is the most common endocrine malignancy. The most sensitive and specific diagnostic tool for thyroid nodule diagnosis is fine-needle aspiration (FNA) biopsy with cytological evaluation. Nevertheless, FNA biopsy is not always decisive leading to “indeterminate” or “suspicious” diagnoses in 10 %–30 % of cases. BRAF V600E detection is currently used as molecular test to improve the diagnosis of thyroid nodules, yet it lacks sensitivity. The aim of the present study was to identify novel molecular markers/computational models to improve the discrimination between benign and malignant thyroid lesions.

**Methods:**

We collected 118 pre-operative thyroid FNA samples. All 118 FNA samples were characterized for the presence of the BRAF V600E mutation (exon15) by pyrosequencing and further assessed for mRNA expression of four genes (KIT, TC1, miR-222, miR-146b) by quantitative polymerase chain reaction. Computational models (Bayesian Neural Network Classifier, discriminant analysis) were built, and their ability to discriminate benign and malignant tumors were tested. Receiver operating characteristic (ROC) analysis was performed and principal component analysis was used for visualization purposes.

**Results:**

In total, 36/70 malignant samples carried the V600E mutation, while all 48 benign samples were wild type for BRAF exon15. The Bayesian neural network (BNN) and discriminant analysis, including the mRNA expression of the four genes (KIT, TC1, miR-222, miR-146b) showed a very strong predictive value (94.12 % and 92.16 %, respectively) in discriminating malignant from benign patients. The discriminant analysis showed a correct classification of 100 % of the samples in the malignant group, and 95 % by BNN. KIT and miR-146b showed the highest diagnostic accuracy of the ROC curve, with area under the curve values of 0.973 for KIT and 0.931 for miR-146b.

**Conclusions:**

The four genes model proposed in this study proved to be highly discriminative of the malignant status compared with BRAF assessment alone. Its implementation in clinical practice can help in identifying malignant/benign nodules that would otherwise remain suspicious.

**Electronic supplementary material:**

The online version of this article (doi:10.1186/s12885-015-1917-2) contains supplementary material, which is available to authorized users.

## Background

Thyroid cancer, which usually presents as a nodule, accounts for approximately 1 % of all newly diagnosed cancer cases and its incidence is increasing faster than any other cancer types, thus representing one of the most common and clinically worrying malignant tumors of the endocrine system [1]. Papillary thyroid carcinoma (PTC) represents the most frequent typology of thyroid malignancy, with a prevalence of about 90 % of all diagnosed cases [1]. Fine-needle aspiration (FNA) biopsy and subsequent cytological analysis represents the most reliable procedure to date to diagnose thyroid nodules [2, 3]. FNA is highly specific for thyroid cancer; however, it has low sensitivity. In fact, 10 %–40 % of the analyzed nodules are detected as indeterminate lesions, thus creating difficulties for the optimal management of these patients [4]. Moreover, only 10 %–30 % of indeterminate thyroid nodules that are surgically resected are confirmed to be malignant [5, 6]. As result, most diagnostic surgeries are performed for benign thyroid nodules. Conversely, patients who have undergone a surgical lobectomy and are found to have a tumor larger than 1 cm, may require a second surgery to remove the remaining thyroid lobe [7, 8], thereby creating an important gap in the clinical decision pathway for thyroid nodules. Clearly, additional diagnostic markers are needed to guide the management of patients with indeterminate thyroid nodules. In the past few years, significant progress has been made in developing molecular markers for clinical use in FNA specimens, such as gene mutation panels and gene expression classifiers [8], but none of these have yet to be accepted as an integral part of the diagnostic tools for clinicians and cytopathologists. BRAF V600E mutation is one the best known and studied prognostic markers for the diagnosis of PTC. The genetic characterization of BRAF status leads to an increase of preoperative diagnostic accuracy up to 20 %–30 % [9, 10]. Nevertheless it stills generates a percentage of suspicious papillary thyroid carcinoma (SPTC) and indeterminate follicular proliferation (IFP) diagnoses. This occurs because some malignant tumors do not have the BRAF V600E mutation, confirming the necessity of finding other molecular markers able to provide a more accurate diagnosis [11]. Few papers have investigated the role of KIT in thyroid cancer as a possible new tumor marker. The KIT gene (CD117) codes for a type III tyrosine-kinase receptor activated by stem cell factor (SCF). Aberrations in KIT expression and signaling, including over-expression or reduced/absent expression, have been characterized in several tumors, such as gastrointestinal stromal tumors, breast cancer, and thyroid carcinoma [12–15], but the role of KIT in human neoplasia is not fully cleared understood. In 2004, Mazzanti et al. identified KIT, from a panel of a thousand genes, as one of the most significant down-regulated gene in PTC compared with benign lesions [16], and in 2012 Tomei et al. showed that KIT was statistically down-regulated in FNA of PTC versus FNA of benign lesions [11]. Next, Tomei et al. showed that the addition of KIT expression increased the diagnostic accuracy of about 15 % compared with cytology-based analysis, but still left a percentage of indeterminate samples [17]. Thus, the same authors determined the diagnostic utility of a nine gene (KIT, SYNGR2, C21orf4, Hs.296031, DDI2, CDH1, LSM7, TC1, and NATH) assay to distinguish benign malignant thyroid neoplasms with a predictive power of 80 % [17]. As miRNAs have been reported to be deregulated in thyroid cancer [18], and they have been shown to function both as tumor suppressors and oncogenes [19], we decided to assess the prediction value of two miRNAs targeting the KIT gene; namely, miR-146b and miR-222. We included in the model the expression of KIT (which has been shown to have the highest prediction value in our previous studies) as well as the TC-1 gene, which is related to thyroid cancer. TC-1 is implicated in the proliferation of cancer cells by regulating Wnt/β-catenin signaling pathways [20–23]. Several studies have shown that this protein is more expressed in thyroid cancers than benign nodules, and the potential use of the TC1 gene expression as a marker of malignancy in thyroid nodules is also shown in the literature [24]. MiR-222 and miR-146b have been shown to be up-regulated at least 10-fold in classic variants of PTC compared with normal thyroid tissue [25]. Several studies have been performed to analyze the utility of miRNAs to differentiate benign from malignant thyroid nodules [26, 27], but few have been performed on FNA indeterminate thyroid lesions [28] or have built miRNA-based predictive models [25]. Since the presence of BRAFV600E assures the malignancy of the thyroid nodule, whereas wild-type BRAF cannot determine a specific diagnosis by itself, we aimed at the evaluation, by quantitative polymerase chain reaction (qPCR) and a computational model, of the expression signature of four genes as a new genetic model to be added to the routine BRAF diagnostic test. We propose this model when BRAF is wild-type in order to improve FNA diagnostic accuracy, especially for the nodules that would otherwise remain suspicious. Our four-gene model was characterized by a lower number of molecular markers compared with the previously developed models, resulting in more practical and usefulness at a clinical level.

## Methods

### FNA samples

Preoperative thyroid FNA slides of 118 thyroid nodules, from as many patients, were collected by an experienced cytopathologist of the Division of Surgical, Molecular and Ultrastructural Pathology, Santa Chiara Hospital, Pisa. The cytology cases included in this study referred to patients who had a thyroidectomy with examination according to standard histological criteria, and all patients had one FNA sample of the lesion. For ethical reasons, we only used cases with extra slides per patient, and representative thyroid cells on the slides, selected by senior cytopathologists, were used to perform molecular analysis.

### Ethics

Prior to the collection of thyroid cells, all patients verbally gave the informed consent to use their cells for research purposes if the collected specimens met specific requirements in terms of diagnosis (e.g. type of lesion) and eligibility (e.g. cytology cases with extra slides per patient). Verbal consent was preferred due to the extremely high number of patients with nodular thyroid pathology every year, the majority of whom are usually willing to donate their samples for research purposes, and the limited number of cases that finally met the criteria of the study. Very few patients are unwilling to provide cells, thus they were asked to sign a non-consent form if consent was not provided, the resulting procedure is easier to manage.

Verbal consent accelerated the cell collection process, reduced paperwork and promoted time efficiency. The study and both verbal consent/written non-consent procedures were approved by the Internal Review Board of the University of Pisa.

### Diagnosis

Histological diagnosis was used to assess malignancy or benignity of all lesions. Criteria used in the cytological diagnosis were smear background, cell shape, cellular arrangements, nuclear/cytoplasmic features, presence of nucleoli, and mitosis, as previously reported [17, 29]. The histological diagnosis of the samples (118) was PTC in 70 cases, and the cytological diagnosis was PTC in 41 cases, SPTC in 19, and IFP in 10 (Table 1). The histological diagnosis in the remaining samples identified 20 benign nodules and 28 IFP (Table 1).

**Table 1:** Histological, cytological, and molecular diagnosis of 118 thyroid nodules

HD	CD		BRAF	
PTC (70)		n	WT	V600E
	PTC	41	15	26
	IFP	10	10	0
	SPTC	19	9	10
BN (48)	IFP	28	28	0
	BN	20	20	0

*HD* histological diagnosis, *CD* cytological diagnosis, *PTC* papillary thyroid carcinoma, *SPTC* suspicious for PTC, *CP* papillary carcinoma, *IFP* indeterminate follicular proliferation, *BN* benign nodule, *WT* wild-type

### DNA and RNA extraction

The slides were kept in xylene until the slide coverslips were detached. Slides were then hydrated in a graded series of ethanol baths, then washed in distilled H_2_O, and finally air-dried. DNA extraction was performed following the manufacturing instructions of a commercial kit (Nucleospin; Macherey-Nagel, Düren, Germany). A modification was added to the first step: 50 % of the lysis solution with no Proteinase K was initially poured on the slides to scrape off the cytological stained sample using a single-edged razor blade. RNA extraction was performed by using a commercial kit (High Pure RNA Paraffin kit, Roche, Indianapolis, IN, USA) according to the manufacturer’s instructions and adding of the same modification step as for DNA extraction. The quality and amount of extracted DNA/RNA was evaluated by NanoDrop 1000 spectrophotometer (Thermo Scientific, Wilmington, DE, USA). RNA was treated with DNase Ι recombinant, RNase-free (Roche, Indianapolis, IN, USA). RNA was reverse-transcribed in a final volume of 20 μL by means of the manufacturer’s instructions of a commercial kit (RevertAid First Strand cDNA synthesis kit, Thermo Scientific, Wilmington, DE, USA).

### miRNA extraction from FNA samples and miRNA expression assay by reverse-transcriptase PCR

Purification of miRNA was performed by using miRNeasyMini Kit (Qiagen, Valencia, CA) according to the manufacturer’s instructions. Quantitative reverse transcription (RT) was performed using miScript II RT Kit, which is an integral component of the miScript PCR System for miRNA detection and quantification (Qiagen, Valencia, CA). cDNA generated from the miScript II RT Kit was used as a template for real-time PCR with the miScript SYBR Green PCR Kit with miRNA specific primers for miR-146b and miR-222 (Qiagen, Valencia, CA). qPCR was run on an Rotor-Gene 6000 (Corbett, Life Science, Sydney, Australia), under the following cycling conditions: 1 cycle at 95 °C for 15 min, 40 cycle at 94 °C for 15 s, 55 °C for 30 s, and 70 °C for 30 s. After 40 cycles, a melting curve was generated by slowly increasing (0.1 °C/s) the temperature from 55 °C to 99 °C, while measuring fluorescence. Samples were detected in triplicate and relative expression levels were calculated using U61 small nuclear RNA (SNORD61, Qiagen, Valencia, CA) as the endogenous control.

### PCR protocol

PCR was performed in a 30 μL final volume, containing 150 ng of cDNA, 0.05 mMdNTP (Invitrogen, Carlsbad, CA, USA), 2.5 ng/μL of each primer (Invitrogen, Carlsbad, CA, USA), 1.5 mM MgCl2, 1x PCR Gold Buffer, and 0.75U AmpliTaq Gold (Applied Byosistems, Foster City, CA, USA). PCR was performed on a 9700 GenAmp PCR System (Applera Corporation, Foster City, CA, USA) under the following cycling conditions: 94 °C for 7 min; 40 cycles at 94 °C for 45 s, 60 °C for 45 s, 72 °C for 1 min, and final step at 72 °C for 10 min.

### Gene expression real-time PCR assay

We used q-real-time PCR to analyze the mRNA expression levels of KIT and TC1 by Rotor-Gene 6000 real time rotary analyzer (Corbett, Life Science, Sydney, Australia) following the manufacturing instructions. A first PCR (see PCR protocol) was performed on control KIT and TC1 expressing samples, then the PCR products were purified by using GeneEluete™ PCR Clean-Up (Sigma-Aldrich, St Louis, MO, USA) and sequenced on the ABI PRISM 3100 Genetic Analyzer (Applied Biosystem, Foster City, CA, USA) to confirm gene sequence. Finally, they were diluted in a 10-fold series to create the standards for a 10-point standard curve that was run in triplicate. Real-time PCR reactions were performed following the manufacturing instructions of the GoTaqqPCR Master Mix Kit (Promega, Madison, WI, USA) in 25 μL final volume containing 2X GoTaqqPCR Master Mix (Promega, Madison, WI, USA), 0.5 μM of each primer (Invitrogen, Carlsbad, CA, USA), and 5 μL of cDNA. The reaction mixtures were subjected to denaturation 95 °C for 2 min, 40 cycles of amplification at 94 °C for 35 s, 60 °C for 35 s, 72 °C for 60 s, and a final step of 72 °C for 10 min. Standard curves were generated for each gene, including beta 2 microglobulin (B2M) that was used to normalize each gene expression level. Post-amplification fluorescence melting curve analysis for each gene was conducted by gradual ramping up the temperature of 0.1 °C/s from 60 °C to 95 °C. No-template reaction was used as a negative control. The expression of all markers was calculated as the ratio of absolute quantification by standard curve of the gene expression value and B2M expression. We used Primer3 software to design the primers for KIT, TC1, and B2M (primer sequences and annealing temperature are shown in the Additional file 1: Table S1).

### BRAF V600E detection

BRAF V600E mutation status was determined using pyrosequencing; PCR amplification and mutational analysis were performed as described in the Diatech manual Anti-EGFR MoAb response (BRAF status). Briefly, PCR amplification was conducted on “Rotor-Gene 6000” (Corbett, Life Science, Sydney, Australia), and was performed on a 151-base-pair region of exon 15 in the BRAF gene including codon 600. All reaction was conducted according to the following protocol: initial denaturation 95 °C for 3 min, 40 cycles at 95 °C for 30 s, 55 °C for 30 s, 72 °C for 30 s, and a final step of 60 °C for 5 min with Takara Ex Taq (Qiagen, Valencia, CA). PCR amplification was then sequenced by PyroMark Q96 ID system (Qiagen, Valencia, CA). Pyrogram outputs were analyzed with the PyroMark Q96 software (Qiagen) to determine the percentage of mutant vs wild-type alleles according to relative peak height.

### Statistical analyses

Quantitative data are expressed as means ± standard deviation. The differences between expression levels of KIT, TC1, miR-146b and miR-222 were analyzed by Student *t*-test and one-way analysis of variance. A difference was considered significant for a *P*-value < 0.05, and the analyses were performed using Statgraphics Centurion (V. 15, StatPoint, Inc.) and MedCalc (Software for Windows version 12, Mariakerke, Belgium). Biomarker data were used to build Bayesian neural networks (BNNs) and to perform discriminant analysis.

The BNN is a nonparametric statistical method based on probabilistic neural networks [30–32], able to classify cases (FNA samples) into different groups of data (malignant, benign) based on a set of quantitative variables (KIT, miR-222, miR-146b, and TC-1). Briefly, the cases are classified according to an artificial neural network, which consists of four layers: 1) input layer, with *k* neurons representing the *k* input quantitative variables (KIT, miR-222, miR-146b, and TC-1); 2) pattern layer, with *n* neurons representing the *n* cases (FNA samples); 3) summation layer, with *q* neurons representing the *q* possible groups (malignant, benign); and 4) output layer, which assigns a case to one of the *q* groups. In layers 1 and 2, the classifier is trained by estimating a nonparametric probability density function for each group. In layer 3, such densities are combined with *prior probabilities* and *misclassification cost functions* to compute a *score* for each of the possible groups where a case may be assigned. Finally, in layer 4, a case is assigned to the group with the largest score. The discriminant analysis [33–35] is a classical parametric method of classification of cases (FNA samples) into different groups of data (malignant, benign), according to a set of quantitative variables (KIT, miR-222, miR-146b, TC-1). The classification of a case (FNA sample) is based on the combination of *prior probabilities* with *discriminant functions*, which assign a *score* to each group (malignant, benign). The case is then assigned to the group with highest score. The discriminant functions are linear combinations of the quantitative variables (KIT, miR-222, miR-146b, and TC-1), and are derived by maximizing the separation of the groups (malignant, benign) in the data. Discriminant analysis is a parametric method because the quantitative variables are assumed to have a normal distribution, conditionally on the group of belonging. All analyses were performed by using Statgraphics Centurion (V. 15, StatPoint, Inc.). We also measured the area under the curve (AUC) of the receiver operating characteristic (ROC) curve for each gene individually in order to validate the diagnostic accuracy of our molecular computational models (MedCalc Software for Windows version 12, Mariakerke, Belgium). Principal component analysis (PCA) and k-means clustering were conducted as descriptive tools by using a R software codes (“princomp” and “kmeans”, package “stats”) [36]. More specifically, we applied a logarithmic transformation of the data to stabilize the variances of the variables (KIT, miR-146b, miR-222, and TC1), since the PCA is sensitive to the relative scaling of the data.

## Results

### BRAF status characterization

All 118 FNA samples analyzed in this study were molecularly characterized for the presence of the BRAF V600E mutation in exon 15: 36/70 malignant samples carried the V600E mutation, while all 48 benign samples were wild type for BRAF exon 15 (Table 1).

### Quantitative markers of gene expression

**Fig. 1:**
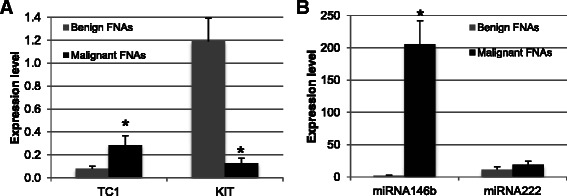
Expression mean for each marker in malignant and benign samples. KIT - TC1 (**a**) and miR-222 - miR-146b (**b**) gene expression levels in benign and malignant thyroid samples

We tested TC1 gene expression in 109 patients (65 malignant, 44 benign), miR-146b and miR-222 expression in 58 FNA smears (41 malignant and 17 benign) and KIT expression in 105 FNA smears (47 malignant and 58 benign) to better understand the relationships between their expression and malignant/benign status. TC1 and miR-146b markers were significantly overexpressed (TC1 *P*-value = 0.04; miR-146b *P*-value = 0.0005) in malignant lesions (TC1 mean = 0.29; miR-146b mean =205.84) compared with benign lesions (TC1 mean = 0.08; miR-146b mean = 2.09). Moreover, miR-222 expression was higher in malignant lesions, but this up-regulation was not statistically significant. Conversely, KIT mRNA expression levels were significantly higher (*P*-value = 0.0006) in benign thyroid tumors (mean = 1.19) compared with malignant tumors (mean = 0.13; Fig. 1).

### Building molecular computational models: classification of malignant and benign samples

In this study, gene expression data were used to build BNNs and to perform discriminant analyses in order to discriminate between benign and malignant disease and predict the probability of thyroid cancer for individual patients. The number of FNA samples taken into account for these analyses was reduced from 118 to 51 to include all the analyzed genes for each patient, and we included malignant samples carrying a BRAF mutation as positive control (Table 2). The BNNs classifier made up of KIT, TC1, miR-222, miR-146b on 51 FNA samples (38 malignant and 13 benign; Table 2), resulted in a predictive power of 94.12 %. It is interesting to note that this model correctly classified 95 % of the samples in the malignant group and 92.31 % of the samples in the benign group (Table 3). The predictive power of KIT, TC1, miR-222, miR-146b expressions to discern malignant from benign lesions was also confirmed by means of discriminant analysis that showed a predictive power of 92.16 % (slightly less than BBNs). Also, more importantly, it correctly classified 100 % of the samples in the malignant group and 69.23 % of the samples in the benign group (Table 4, Additional file 2: Table S2). In order to validate the accuracy of the models as predictive tools, we conducted a *blind analysis* on 11 unknown samples, with both discriminant analysis and BNNs. At the end of the analysis, our models diagnosed all the 11 unknown samples in accordance with pathological diagnosis. Discriminant analysis gave a benign probability of 0.1101 and a malignant probability of 0.8898, while BNNs determined 0.0764 and 0.9264, respectively (Tables 5 and 6). The samples correctly classified were diagnosed as SPTC at the cytological level and were moved to the diagnostic group of malignant after pathological diagnosis. Seven of the 11 SPTC samples used in this analysis had BRAF mutations. Therefore, there were four BRAF wild-type patients. Our model assigned these four patients to the malignant group with a probability of 0.9065, 0.8631, 0.7890, 0.9585 by discriminant analysis and 0.999, 0.824, 0.799, 1 by BNNs, respectively (Tables 5 and 6).

**Table 2:** Histological, cytological, and molecular diagnosis of 51 thyroid nodules used in the computation models

HD	CD		BRAF	
PTC (38)		n	WT	V600E
	PTC	22	10	12
	IFP	5	5	0
	SPTC	11	4	7
BN (13)	IFP	7	7	0
	BN	6	13	0

*HD* histological diagnosis, *CD* cytological diagnosis, *PTC* papillary thyroid carcinoma, *SPTC* suspicious for PTC, *CP* papillary carcinoma, *IFP* indeterminate follicular proliferation, *BN* benign nodule, *WT* wild-type

**Table 3:** Classification table of Bayesian neural networks. Predictive power of KIT, TC1, miR-222, and miR-146b for discriminating malignant from benign: among the 51 cases used to train the model, 94.12 % of them were correctly classified

*Actual mal_ben*	*Group size*	*Predicted*	
		Benign	Malignant
Benign	13	12	1
		(92.31 %)	(7.69 %)
Malignant	38	2	36
		(5.26 %)	(94.74 %)

**Table 4:** Classification table of discriminant analysis. Predictive power of KIT, TC1, miR-222, and miR-146b for discriminating malignant from benign FNA. This procedure is designed to develop a set of discriminating functions which can help predict malignant vs. benign status based on the values of other quantitative variables; 51 cases were used to develop a model to discriminate among the two levels of malignant vs. benign; four predictor variables were entered. Amongst the 51 observations used to fit the model, 47 % or 92.16 % were correctly classified

*Actual mal_ben*	*Group size*	*Predicted*	
		Benign	Malignant
Benign	13	9	4
		(69.23 %)	(30.77 %)
Malignant	38	0	38
		(0.00 %)	(100.00 %)

Classification variable: Malignant vs Benign

Independent variables: KIT, TC1, miR-222, miR-146

**Table 5:** Gene model validation test by discriminant analysis. Malignant or benign group allocation probability values for the unknown samples

Unknown samples	Benign probability	Malignant probability	Predicted diagnosis	Cytological diagnosis	Pathological diagnosis	BRAF status
A	0.0700	0.9300	Malignant	SPTC	Malignant	V600E
B	0.0530	0.9470	Malignant	SPTC	Malignant	V600E
C	0.1075	0.8925	Malignant	SPTC	Malignant	V600E
D	0.0177	0.9823	Malignant	SPTC	Malignant	V600E
E	0.1964	0.8036	Malignant	SPTC	Malignant	V600E
F	0.1380	0.8620	Malignant	SPTC	Malignant	V600E
G	0.0935	0.9065	Malignant	SPTC	Malignant	WT
H	0.1369	0.8631	Malignant	SPTC	Malignant	WT
I	0.1458	0.8542	Malignant	SPTC	Malignant	V600E
L	0.2110	0.7890	Malignant	SPTC	Malignant	WT
M	0.0415	0.9585	Malignant	SPTC	Malignant	WT

**Table 6:** Gene model validation test by BNN analysis. Malignant or benign group allocation probability values for the unknown samples

Unknown samples	Benign probability	Malignant Probability	Predicted diagnosis	Cytological diagnosis	Pathological diagnosis	BRAF status
A	0.0302	1.0000	Malignant	SPTC	Malignant	V600E
B	0.0011	1.0000	Malignant	SPTC	Malignant	V600E
C	0.0004	0.9996	Malignant	SPTC	Malignant	V600E
D	0.0000	1.0000	Malignant	SPTC	Malignant	V600E
E	0.3242	0.6758	Malignant	SPTC	Malignant	V600E
F	0.0223	0.9777	Malignant	SPTC	Malignant	V600E
G	0.0009	0.9991	Malignant	SPTC	Malignant	WT
H	0.1759	0.8241	Malignant	SPTC	Malignant	WT
I	0.0847	0.9153	Malignant	SPTC	Malignant	V600E
L	0.2007	0.7993	Malignant	SPTC	Malignant	WT
M	0.0000	1.0000	Malignant	SPTC	Malignant	WT

### Principal component analysis

**Fig. 2:**
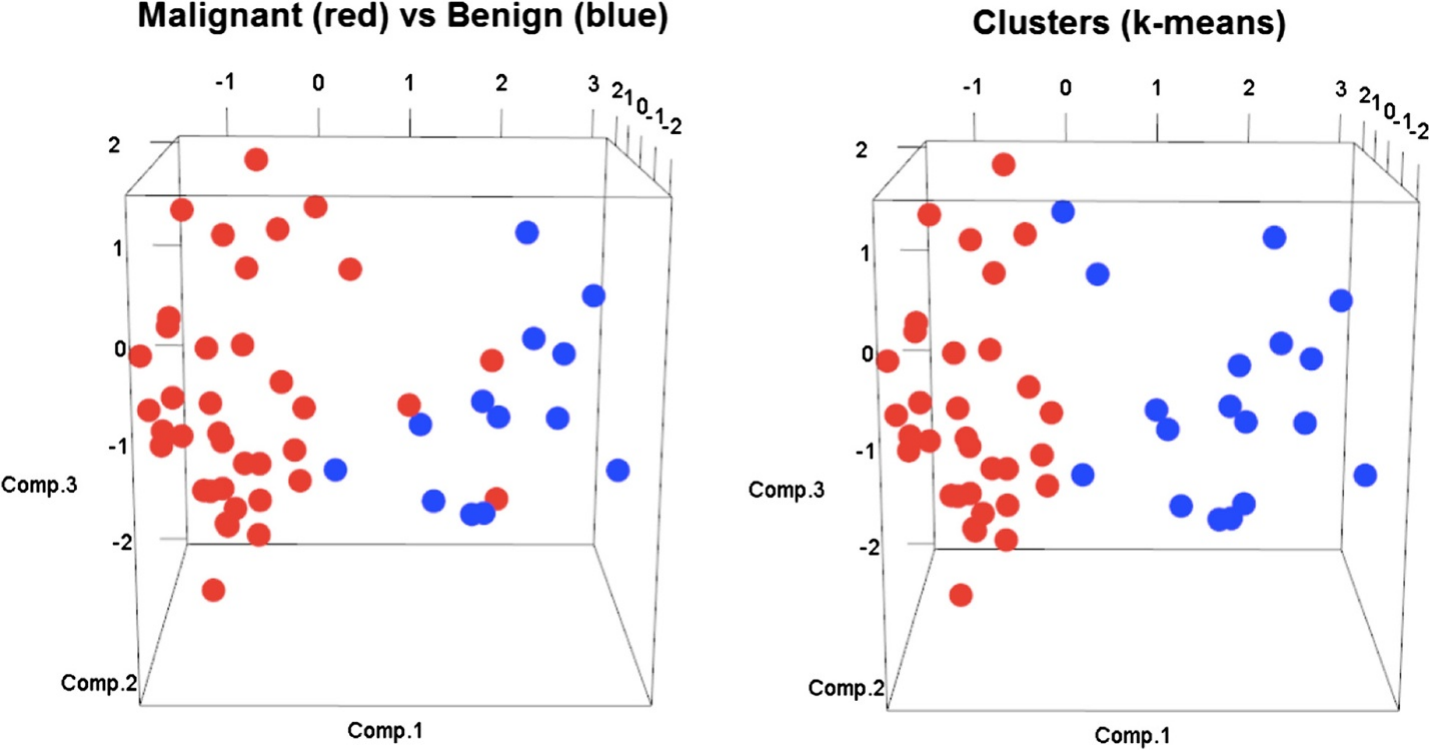
Principal component analysis and k-means clustering. We plot the first three principal components of the space of the four log transformed features TC1, c-KIT, miR-146, and miR-222 in the context of classifying malignant vs benign. The data points in the plots on the left are labeled according to their condition (“Malignant vs Benign”). The plots on the right show the clusters identified by the unsupervised analysis performed via k-means clustering. We can see that the separation induced by the conditions “Malignant vs Benign” approximately reproduces/reflects the intrinsic grouped structure of the data

We next performed PCA in order to visualize in a three-dimensional space the discriminative power of all four markers according to malignant and benign status (Fig. 2). A separation between malignant and benign samples can be visually identified (Fig. 2, left plot). A similar grouped structure was identified by an unsupervised analysis performed via “k-means” clustering (Fig. 2, right plot).

### ROC curve analysis

**Fig. 3:**
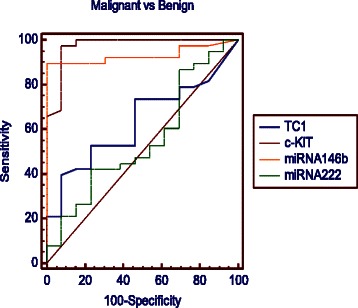
ROC analysis for KIT, TC1, miR-146b, miR-222 for case classification into malignant vs benign. KIT and miRNA146b showed the highest discriminating power (AUC = 0.9). The true positive rate (sensitivity) is plotted as a function of the false positive rate (100-specificity) for different cutoff points. Each point on the ROC plot represents a sensitivity/specificity pair corresponding to a particular decision threshold

In order to determine the model robustness for predicting malignancy in thyroid samples, we finally resorted to ROC curve analyses by individually using the expression of each marker (TC1, KIT, miR-146b, miR-222; Fig. 3, Table 7). Among all markers, KIT and miRNA146b showed the highest AUC (0.9) for malignant versus benign.

**Table 7:** Individual ROC analysis for each marker in malignant vs benign

	Sensitivity	Specificity	AUC	SE	95 % CI	*p*-value
TC1	38.5	92.9	0.634	0.0816	0.487 to 0.764	0.0953
c-KIT*	95.7	88.2	0.973	0.0261	0.883 to 0.998	<0.0001
miR146b*	87.8	100.00	0.931	0.0364	0.824 to 0.983	<0.0001
miR-222	48.8	68.7	0.551	0.0955	0.405 to 0.690	0.9171

*AUC* area under the curve, *SE* standard error, *CI* confidence interval

*P < 0.05

### Association analysis between miRNA146b, miRNA 222, TC1, and KIT gene expression level and BRAF V600E mutation

**Fig. 4:**
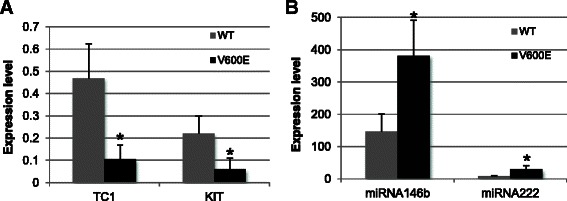
Expression mean for each marker in BRAF WT and V600E malignant samples. KIT - TC1 (**a**) and miR-222 - miR-146b (**b**) expression in BRAF wild-type versus V600E malignant lesions

We investigated the expression of miRNA146b, miRNA 222, TC1, and KIT in only malignant FNAs: there were 41 malignant FNAs with 20/41 carrying the V600E mutation on BRAF exon 15. We found that miR-146b and miR-222 were significantly down-regulated (*P*-value = 0.036; *P*-value = 0.037, respectively) in the malignant samples with wild-type BRAF (mean = 146.57; mean = 8.15, respectively) compared with the malignant group with BRAF V600E (mean = 381.73; mean = 29.59, respectively). The opposite was found for KIT (mean = 0.06 for BRAF V600E; mean = 0.22 for wild-type BRAF, *P*-value = 0.023) and TC1 (mean = 0.10 for BRAF V600E; mean = 0.47 for wild-type BRAF, *P*-value = 0.009), which carried the V600E mutation in 28/47 and 34/65 of malignant samples, respectively (Fig. 4).

## Discussion

The current diagnosis of thyroid nodules, based on FNA cytology, still leads to a significant proportion of indeterminate lesions. In the past few years, several studies have investigated the development of molecular markers to play a diagnostic role in FNA specimens [8]. Nevertheless, the studied genes still have limited diagnostic power owing to the small number of screened patients or because only a few authors tested these markers on indeterminate lesions to conclude a definitive diagnosis; furthermore, there are many contradictory results in the literature [25, 37]. Owing to the lack of useful pre-operative diagnostic biomarkers and in view of acquiring a better understanding of the correct diagnosis of indeterminate lesions, we herein proposed new markers, such as KIT, TC1, miR-146b and miR-222. We found that KIT mRNA expression levels were significantly higher in benign thyroid tumors compared with malignant ones, thereby confirming our previous results [11]. Few papers have suggested to analyze KIT expression on FNA biopsies from benign and malignant thyroid nodules to verify if KIT expression analysis is of clinical interest. Down-regulated KIT expression in thyroid tumors is in contrast with the over-expression of other tyrosine kinase receptors, such as c-RET and c-MET, or oncogenes, such as c-RAS, indicating that the signaling pathways of different tyrosine kinase receptors can control opposite biological mechanisms, or alternatively affect cell proliferation or differentiation in a specific cell type. The KIT ligand, SCF, operates in conjunction with thyroid-stimulating hormone; however, it is not a mitogenic factor in primary thyrocytes cultures [38], which suggests that the SCF/KIT pathway might be involved in thyrocyte differentiation rather than proliferation. By investigating the diagnostic ability of miR-222 and miR-146b in our FNA samples, we showed that miR-146b was significantly over-expressed in malignant lesions, as reported in the literature [25], and that miR-222 expression was also higher in the malignant group compared with the benign group, although this did not reach significance. Since miR-146b is more accurate at differentiating malignant from benign thyroid lesions on FNA, we suggest that FNA miR-146b analysis is a useful adjunct in the management of patients with thyroid nodules. The concomitant increase in the expression of the two miRNAs that target KIT [18, 39] and the decrease in KIT expression in our malignant FNA samples strengthens the choice to use these markers in the diagnosis of nodules. TC1 has been reported to be over-expressed in thyroid cancer compared with benign nodules [24, 40], and according to the literature, we found significant over-expression of TC1 in malignant lesions compared with benign lesions. The exact function of the protein coded by this gene is still unknown, although the overexpression of TC-1 in papillary carcinoma suggests that it may play an important role in thyroid carcinogenesis. Medical diagnoses are progressing quickly as a result of computational advances, for example computation model like discriminant analysis and BNNs, and have been proven to generate better results compared with standard statistical techniques [41, 42]. BNNs and discriminant analyses made up of KIT, TC1, miR-222, and miR-146b performed on data collected from FNA samples showed a very strong predictive value (94.12 % and 92.16 %, respectively) for discriminating malignant from benign patients. It is noteworthy that discriminant analysis showed a correct classification of 100 % of the samples in the malignant group, and 95 % by BNN (Tables 3 and 4). Based on the discriminant analysis, the predicted probability of disease resulted to range between 85 % and 100 % for almost all disease cases. No classification errors occurred when the predicted probability of the disease was higher than 85 %; hence, the use of the four genes as a case classifier strengthens their importance as preoperative predictors of diagnosis of thyroid nodules (Additional file 2: Table S2). Of note, miR-222 relevantly contributed to strengthening the discriminative power, even if it was not a significant marker itself for the discrimination of malignant from benign samples. Both the models were validated using 11 unknown samples. Referring to the standard pathological diagnosis conducted by clinical pathologists, they lead to an accurate diagnosis (Tables 5 and 6). In particular, the samples that were correctly classified were diagnosed as indeterminate samples (SPTC) at the cytological level; 7 of the 11 SPTC samples used in this analysis were BRAF mutated. Therefore, there were four patients left out that even after BRAF mutational analysis remained SPTC. Our model assigned these four patients to the malignant group, with a high probability on both discriminant analysis and by BNN. Our data demonstrate that our model can make the diagnosis of malignancy with more certainty than a surgeon. It is important to point out that SPTC lesions are often very difficult to diagnose, and in this study we developed a molecular approach that is able to correctly classify with 100 % certainty the unknown SPTC samples as malignant. Because our markers panel is 100 % sensitive for malignant pathology of indeterminate FNA lesions, it would be reasonable to recommend a total thyroidectomy if malignancy is predicted. In order to visualize in a three-dimensional space the discriminative power of all the four markers, we applied a PCA to the benign and malignant samples. We obtained an overall separation among them according to the expression of the four markers used in the study, which confirmed that the four markers together discriminate between benign and malignant status. Using the dataset from the computational model and the PCA analysis, we also performed ROC analysis in order to optimize the model for negative and positive predictive values in our thyroid cohort. The ROC curve of c-KIT and miRNA146b had a high diagnostic accuracy for FNA samples, nearing 100 %; therefore, they alone and in combination can be used to distinguish between malignant and benign nodules. On the other hand, the ROC curve of TC1 had high specificity (92.9), which means that when TC1 is over-expressed in our samples it has a high probability to correctly identify the samples as malignant with a low risk of false positives, but it had low sensitivity (38.5). Therefore, when the value of TC1 is low there is a high probability to have a false benign result. Further analyses revealed that the expression levels of the four genes are also significantly associated with the molecular status of the BRAF gene. As a matter of fact, as shown in Fig. 4, in the BRAF mutated group, the down-regulation of KIT and up-regulation of miR-146b and miR-222 are indicative of a more aggressive behavior reflecting the same trend between benign and malignant lesions. On the other hand, TC1 expression levels have the opposite behavior from what is observed earlier between the malignant and benign lesions, indicative therefore of a mutual exclusive malignancy driving with respect to BRAF V6000E. Our hypothesis is that when the malignant transformation is driven by mutated BRAF, TC1 has no influence on the transformation; however, when BRAF is wild-type, TC1 has a major role in neoplastic transformation. These results shows how the presence of the BRAF V600E mutation is accompanied by a specific genetic scenario in which sets of genes discriminate the mutational and wild-type status, supporting the hypothesis of higher tumor aggressiveness associated with the BRAFV600E mutation.

## Conclusions

In conclusion, herein we were able to develop a statistical model that accurately differentiates malignant from benign indeterminate lesions on thyroid FNAs using a panel of two miRNAs and two genes (miR-146b, miR-222, KIT, and TC1). We suggest the use our four-gene model as a further step in the diagnosis of suspicious nodules in clinical cases with an indeterminate cytological analysis and wild-type BRAF molecular marker.

## Additional files

Additional file 1: Table S1. Set of primers used for genes analyses. (PDF 177 kb)
Additional file 2: Table S2. Scores list of Discriminant Analysis. (PDF 164 kb)

## Abbreviations

AUC: Area under the curve
BNN: Bayesian neural network
FNA: fine-needle aspiration
PCA: principal component analysis
PCR: Polymerase chain reaction
PTC: papillary thyroid cancer
q: quantitative
ROC: receiver operating characteristic
RT: reverse transcriptase
