# Supplementary material for: The combination of four molecular markers improves thyroid cancer cytologic diagnosis and patient management
Source: BMC Cancer. 2015 Nov 19;15:918. doi: 10.1186/s12885-015-1917-2 (PMC4652365; doi:10.1186/s12885-015-1917-2)
Supplement: Additional file 1: Table S1. — Set of primers used for genes analyses. (PDF 177 kb) [file 12885_2015_1917_MOESM1_ESM.pdf]

Table S1: set of primers used for genes analyses.

PRIMERS FOR EXPRESSION STUDY		
NAME	SEQUENCE (5'-3')	Annealing temperature (C^0)
KIT F	GCACCTGCTGCTGAAATGTATGACATAT	60
KIT R	TTTGCTAAGTTGGAGTAAATATGATTGG	60
TC1 F	AAATCTTCTGACTAATGCTAAAACG	60
TC1 R	TTATTGTTGCATGACATTTGC	60
B2M F	CATTCCTGAAGCTGACAGCATTC	60
B2M R	TGCTGGATGACGTGAGTAAACC	60
