# Supplementary material for: The combination of four molecular markers improves thyroid cancer cytologic diagnosis and patient management
Source: BMC Cancer. 2015 Nov 19;15:918. doi: 10.1186/s12885-015-1917-2 (PMC4652365; doi:10.1186/s12885-015-1917-2)
Supplement: Additional file 2: Table S2. — Scores list of Discriminant Analysis. (PDF 164 kb) [file 12885_2015_1917_MOESM2_ESM.pdf]

Table S2: Scores list of Discriminant Analysis.

The classification table lists the two highest scores amongst the classification functions for each of the 51 observations used to fit the model, as well as for any new observations. The discriminating function with *p*-value less than 0.05 is considered statistically significant at the 95.0% confidence level.

Row	*Actual Group*	*Highest Group*	*Highest Value*	*Squared Distance*	*Prob.*	*2nd Highest Group*	*2nd Highest Value*	*Squared Distance*	*Prob.*
1	1	1	-0.996281	0.0704307	0.8839	0	-3.02616	4.13018	0.1161
2	1	1	-1.24553	0.126248	0.8610	0	-3.06878	3.77276	0.1390
3	1	1	-1.13294	0.995233	0.5860	0	-1.4804	1.69015	0.4140
4	1	1	-0.146934	0.0000101817	0.9329	0	-2.77925	5.26464	0.0671
5	1	1	0.444883	0.0586689	0.9607	0	-2.7513	6.45104	0.0393
6	1	1	0.959299	0.116463	0.9684	0	-2.46447	6.964	0.0316
7	1	1	-0.0437119	0.0456246	0.8956	0	-2.19258	4.34336	0.1044
8	1	1	-0.35565	0.0060648	0.9213	0	-2.81636	4.92749	0.0787
9	1	1	-0.772161	0.236599	0.8208	0	-2.29419	3.28065	0.1792
10	1	1	-0.909852	0.173664	0.8432	0	-2.59199	3.53794	0.1568
11	1	1	-0.978737	0.0830749	0.8784	0	-2.95613	4.03787	0.1216
12	1	1	-0.0884061	0.000111853	0.9318	0	-2.70375	5.23081	0.0682
13	1	1	2.95808	0.500472	0.9861	0	-1.30703	9.0307	0.0139
14	1	1	4.42059	3.26349	0.9989	0	-2.36984	16.8443	0.0011
15	1	1	-1.18884	0.124011	0.8618	0	-3.01936	3.78505	0.1382
16	1	1	0.893555	0.0104766	0.9466	0	-1.98127	5.76013	0.0534
17	1	1	-0.768977	0.0310464	0.9033	0	-3.00378	4.50064	0.0967
18	1	1	1.50373	0.117109	0.9685	0	-1.92221	6.96899	0.0315
19	1	1	-1.33819	0.566862	0.7129	0	-2.24791	2.38631	0.2871
20	1	1	-0.644114	0.0165925	0.9124	0	-2.98779	4.70395	0.0876
21	1	1	-1.00743	0.0899351	0.8755	0	-2.95803	3.99113	0.1245
22	1	1	-0.614894	0.0297171	0.9041	0	-2.85845	4.51684	0.0959
23	1	1	-0.83946	0.0455392	0.8956	0	-2.98879	4.34419	0.1044
24	1	1	1.28208	0.247275	0.9777	0	-2.50012	7.81169	0.0223
25	1	1	-0.355491	0.000281892	0.9309	0	-2.95656	5.20242	0.0691
26	1	1	-1.23095	0.141747	0.8550	0	-3.00554	3.69093	0.1450
27	1	1	-0.809608	0.0373955	0.8998	0	-3.00494	4.42805	0.1002
28	1	1	1.39246	0.073457	0.9631	0	-1.86993	6.59823	0.0369
29	1	1	2.73132	1.06855	0.9934	0	-2.28344	11.0981	0.0066
30	0	0	10.64	14.3598	1.0000	1	-0.706503	37.0528	0.0000
31	0	0	-0.462702	1.20031	0.5306	1	-0.585055	1.44501	0.4694
32	0	0	0.644535	0.145789	0.8535	1	-1.11781	3.67048	0.1465
33	0	0	7.67354	5.9752	0.9997	1	-0.582584	22.4875	0.0003
34	0	*1	-1.19548	1.03641	0.5746	0	-1.496	1.63745	0.4254
35	0	0	8.1872	7.8541	0.9999	1	-0.891717	26.0119	0.0001
36	0	0	0.546552	0.166674	0.8457	1	-1.15506	3.56989	0.1543
37	0	*1	-1.33338	0.177548	0.8418	0	-3.00488	3.52053	0.1582
38	0	*1	-1.31058	0.713334	0.6680	0	-2.00964	2.11145	0.3320
39	1	1	-0.965599	0.0847317	0.8777	0	-2.93642	4.02638	0.1223
40	0	0	2.60575	0.00221143	0.9263	1	0.0741535	5.06541	0.0737
41	1	1	0.0980452	0.0137514	0.9483	0	-2.81104	5.83193	0.0517
42	1	1	0.122645	0.00124895	0.9382	0	-2.5982	5.44295	0.0618
43	1	1	-0.62535	0.0207164	0.9096	0	-2.93429	4.6386	0.0904
44	1	1	2.17636	0.60257	0.9881	0	-2.24687	9.44902	0.0119
45	1	1	-0.715366	0.0282095	0.9050	0	-2.9691	4.53569	0.0950
46	1	1	1.13048	0.33086	0.9813	0	-2.8308	8.25342	0.0187
47	1	1	-1.31618	0.161647	0.8476	0	-3.03204	3.59337	0.1524
48	1	1	-0.200962	0.00208922	0.9265	0	-2.73559	5.07134	0.0735
49	0	0	-0.517045	0.922166	0.6066	1	-0.950252	1.78858	0.3934
50	0	0	0.835918	0.100527	0.8711	1	-1.07523	3.92283	0.1289
51	0	*1	-1.31641	1.16719	0.5393	0	-1.47373	1.48184	0.4607

* = incorrectly classified.0 benign, 1 malignant.
